# Chemopreventive Potential of Artemisinin and *Rubus occidentalis* in the Progression of Oral Leukoplakia to Oral Cancer: A Preclinical Murine Study

**DOI:** 10.3390/ijms26178120

**Published:** 2025-08-22

**Authors:** Maria Leticia de Almeida Lança, Nathan Steven Cezar da Conceição, Isabella Souza Malta, Daniela Oliveira Meneses, Luciana Yamamoto Almeida, Estela Kaminagakura

**Affiliations:** Department of Bioscience and Oral Diagnosis, Institute of Science and Technology, University of São Paulo State, Avenue Engenheiro Francisco José Longo, 777, São José dos Campos, São Paulo 12245-000, Brazil; leticia.lanaa@unesp.br (M.L.d.A.L.); nathan.stevan@unesp.br (N.S.C.d.C.); isabella.malta@unesp.br (I.S.M.); daniela.meneses@unesp.br (D.O.M.); luciana.yamamotodealmeida@nih.gov (L.Y.A.)

**Keywords:** oral leukoplakia, squamous cell carcinoma of the head and neck, chemoprevention, Artemisinins, Black Raspberry, 4-Nitroquinoline-1-oxide

## Abstract

Oral leukoplakia (OL) is the most common potentially malignant oral disorder, with variable risk of progression to oral squamous cell carcinoma (OSCC). This study evaluated the chemopreventive and immunomodulatory potential of Artemisinin (ART) and *Rubus occidentalis* (RO), alone or combined (ARO), in a 4NQO-induced murine model. Mice received 4NQO (100 µg/mL) in drinking water, and treatments began at week 8. Animals were euthanized at weeks 12 and 16 for histological, apoptotic (caspases-3, -8, -9; calreticulin), inflammatory (IL-1β, IL-10, HMGB1), and immune (CD8, CD68, CD56, IFN-γ, GM-CSF) marker analyses. RO-treated animals showed delayed malignant transformation, with no carcinomas at week 16 and increased expression of caspase-9, calreticulin, HMGB1, IFN-γ, and GM-CSF, indicating transient activation of antitumor immune responses. ART-treated mice showed increased CD68 and reduced CD56 expression, suggesting an immunosuppressive profile and higher carcinoma incidence. The ARO combination did not improve outcomes beyond ART alone. These findings support the immunomodulatory and pro-apoptotic effects of RO in delaying OL progression, highlighting its chemopreventive potential. ART showed limited benefit under current conditions, warranting further investigation into dose optimization and synergistic strategies.

## 1. Introduction

Oral Potentially Malignant Disorders (OPMDs) are alterations of the oral mucosa associated with a statistically increased risk of malignant transformation [1]. Among them, oral leukoplakia (OL) is the most prevalent, with an estimated rate ranging from 1.36% to 2.6% in the general population [2,3,4]. OL is a predominantly white plaque of questionable risk, having excluded (other) known diseases or disorders that do not carry an increased cancer risk [1]. Its development is frequently associated with exposure to carcinogenic agents, particularly tobacco, being approximately six times more common in smokers than in non-smokers [2]. However, idiopathic cases without identifiable causal factors have also been reported [3]. OL generally affects individuals from the fourth decade of life onwards and is more prevalent among men [3,5]. The presence and degree of epithelial dysplasia are key determinants of malignant transformation risk, with moderate to severe dysplastic lesions exhibiting a higher potential for progression to carcinoma [1]. The presence and degree of epithelial dysplasia influence the risk of malignant transformation of OL [6]. Due to the limited efficacy of conventional treatments, especially surgical excision, in preventing recurrence and malignant transformation, alternative strategies such as chemoprevention have emerged as promising therapeutic avenues [7]. Both systemic and topical chemopreventive agents have been explored to enhance treatment outcomes and mitigate the progression of OL to oral cancer [8].

Among the agents under investigation, Artemisinin (ART) and its derivatives, such as dihydroartemisinin, artesunate, and artemether, have demonstrated remarkable anticancer activity against various cancer types [9]. These effects include inhibiting cell proliferation, migration, and invasion; suppressing angiogenesis and anaerobic glycolysis; and inducing cell cycle arrest, autophagy, and cell death in addition to an immunomodulatory role [10,11]. The antitumor mechanisms of ART are mainly attributed to the induction of oxidative stress in malignant cells, with increased generation of reactive oxygen species, which leads to DNA damage, mitochondrial dysfunction, and activation of the caspase-3-dependent apoptotic pathway [12,13,14]. Beyond apoptosis, ART can also trigger non-apoptotic forms of cell death, such as ferroptosis and autophagy. Successful combinations of ART with chemotherapeutics or phytochemicals further reinforce its potential as an adjuvant in cancer therapy [15].

*Rubus occidentalis* L. (RO), known as black raspberries, has also demonstrated anticancer potential, being rich in bioactive compounds such as anthocyanins, ellagitannins, and ascorbic acid [16]. Their therapeutic effects occur through multiple mechanisms, including antioxidant activity, DNA damage repair, epigenetic modulation, apoptosis induction, cell cycle interference, glycolysis inhibition, antiangiogenic action, tumor immunity regulation, and anti-inflammatory effects [17,18]. Additionally, RO promotes CD8^+^ cytotoxic T cell activity and reduces regulatory T cell recruitment, suggesting a role in immunogenic cell death and strengthening the antitumor immune response [19].

Considering the therapeutic potential of these compounds, we investigated whether ART, RO, and their combination could prevent OL progression to oral squamous cell carcinoma (OSCC) through immunomodulation. Therefore, this study aimed to evaluate the effects of these agents during the early stages of 4-nitroquinoline 1-oxide (4NQO)-induced OSCC development in mice by assessing markers of apoptosis, inflammation, and immune response.

## 2. Results

### 2.1. Weight and Toxicity Analysis

**Figure 1:**
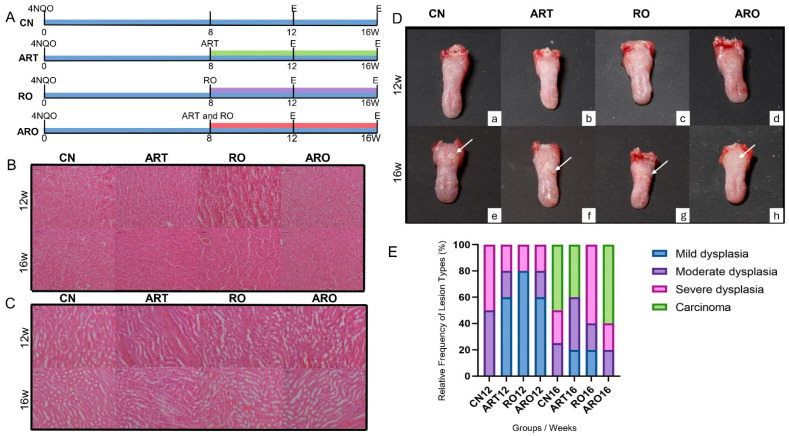
4NQO-induced oral carcinogenesis and systemic evaluation of treatment safety in mice. (**A**) Experimental design and timeline. All animals were acclimated for 15 days before exposure to 4NQO in drinking water. Treatments began at week 8, and animals were euthanized at weeks 12 or 16. Experimental groups included ART, RO, and ARO. The CN received only 4NQO. (**B**) Representative H&E–stained sections of liver from treated and control animals. The liver architecture was preserved, with no histopathological evidence of inflammation, necrosis, or structural alterations. (**C**) Representative H&E–stained sections of kidney from treated and control animals. Renal structures, including glomeruli and tubules, appeared normal, with no signs of degeneration, necrosis, or inflammatory infiltration. These findings indicate the absence of systemic toxicity from the treatments. (**D**) Macroscopic images of mouse tongues by group and time point, illustrating surface changes. **Panels a**–**d** show representative tongues with more opaque and whitish surfaces. In the CN group (**panel e**), the arrow points to a prominent white nodule. In the ART (**panel f**) and RO (**panel g**) groups, the arrows indicate erythematous regions. In the ARO group (**panel h**), the arrow highlights a verrucous area. (**E**) Bar graphs showing the relative frequency of histological diagnoses (mild, moderate, and severe dysplasia; carcinoma) in each group. At week 12, only treated groups presented with dysplasia, predominantly mild. By week 16, lesion progression was evident in all groups, with carcinoma observed in CN, ART, and ARO. Notably, RO-treated animals exhibited no carcinomas but a higher incidence of severe dysplasia.

Throughout the experiment, the animals exhibited normal behavior, showed no signs of distress, and maintained stable body weight, with no significant variations exceeding 10%. Kidneys and livers showed no cellular alterations or signs of toxicity. No significant changes were observed in the kidneys across all groups and the time points analyzed. Renal corpuscles and glomeruli maintained their normal size without evidence of atrophy. Furthermore, no hemorrhagic foci or inflammatory cell infiltration was detected. In the liver, inflammatory infiltrates, steatosis, hepatocyte ballooning degeneration, fibrosis, and hemorrhagic areas were investigated, but none of these alterations were identified in the analyzed groups (Figure 1B,C).

### 2.2. Macroscopic Analysis

By the 12th week, well-defined whitish plaques with a smooth or slightly rough surface emerged. Over time, these lesions became more irregular, with undefined borders and scattered reddish areas (Figure 1D). In the CN group, panel e, a prominent white nodule compatible with carcinoma is observed, indicated by the arrow. In the ART group, panel f, and in the RO group, panel g, the arrows indicate areas that became erythematous. In the ARO group, panel h, a well-defined verrucous area is noted. These macroscopic changes illustrate the diversity of lesion patterns observed throughout the study.

### 2.3. Histological Analysis

**Figure 2:**
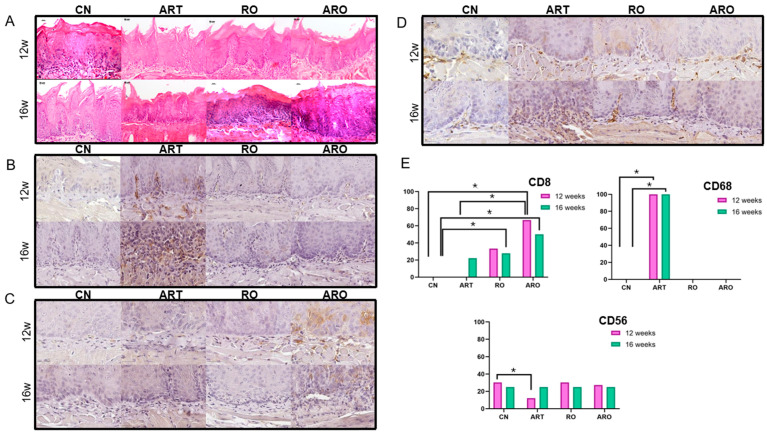
Histological and immunohistochemical analyses. (**A**) Histological sections of the tongue over time, showing mild, moderate, and severe dysplasia, as well as oral squamous cell carcinoma in different groups at different time points, stained with H&E. (**B**–**D**) Representative images of immunohistochemical staining for CD8, CD68, and CD56, respectively, in experimental and control groups. (**E**) Relative frequency of CD8^+^, CD68^+^, and CD56^+^ cells across experimental groups at weeks 12 and 16. Data represent normalized expression within each time point, allowing for comparison between treatment groups (*p* < 0.05). * Statistically significant *(p* < 0.05).

Abnormal morphological alterations varied among groups over time. At week 12, no animals in the control group (CN) exhibited mild dysplasia, whereas all treated groups (ART, RO, and ARO) predominantly presented with mild dysplasia, with the highest frequency observed in group RO (80%). Moderate and severe dysplasia were also identified at lower frequencies in groups ART (20% and 20%, respectively), RO (0% and 20%), and ARO (20% and 20%), with no carcinomas detected in any group at this stage. By week 16, lesion progression was observed in all groups. In the control group, two animals (50%) developed carcinoma. Group ART showed an increase in severity, with two mice (40%) of the animals presenting with carcinoma. In group ARO, three animals (60%) developed carcinoma. In contrast, group RO did not present any carcinomas at this time point and instead showed an increase in severe dysplasia cases (60%) (Figure 1E and Figure 2A).

### 2.4. Immunohistochemistry

To explore the immune response during LO progression and treatment, markers for T lymphocytes (CD8), macrophages (CD68), and Natural Killer cells (CD56) were characterized. Immune cells were variably distributed in the epithelium and lamina propria. The number of CD8^+^ cells analysis revealed significant differences among experimental groups over time. At week 12, the ARO group showed significantly higher counts compared to CN and ART (*p* < 0.001). At week 16, ARO maintained elevated counts compared to CN (*p* < 0.001), and RO differed from CN (*p* = 0.003), suggesting a delayed effect of this treatment. The number of CD68^+^ cells was significantly higher in group ART at weeks 12 and 16 (*p* ≤ 0.010). The number of CD56^+^ cells was reduced in group ART at week 12 (*p* < 0.001), whereas CN, RO, and ARO did not differ from one another. At week 16, all groups exhibited the same number of positive cells. Immunohistochemistry images and statistical data are presented in Figure 2B–E.

### 2.5. ELISA

**Figure 3:**
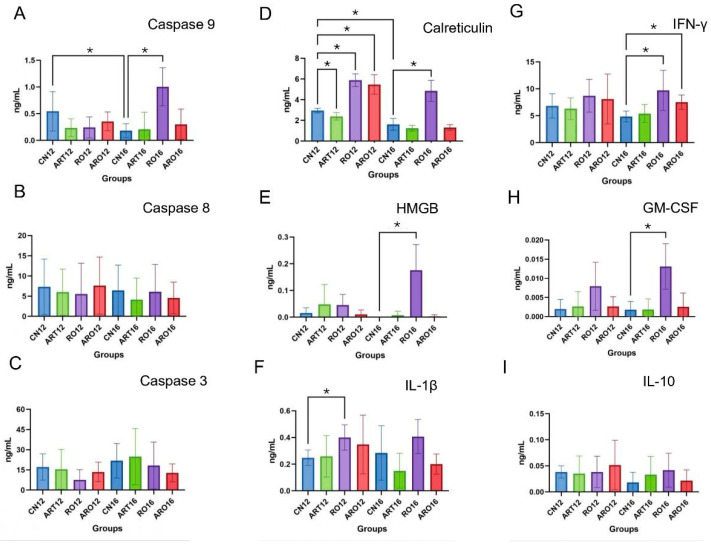
Immunological profiling by ELISA. Protein levels of Caspase-9 (**A**), Caspase-8 (**B**), Caspase-3 (**C**), Calreticulin (**D**), HMGB1 (**E**), IL-1B (**F**), IFN-γ (**G**), GM-CSF (**H**), and IL-10 (**I**) in tongue tissues of mice exposed to 4NQO and treated with Artemisinin (ART), *Rubus occidentalis* extract (RO), or the combination of both (ARO), with the control group (CN) receiving no treatment, at 12 and 16 weeks (*p* < 0.05). * Statistically significant (*p* < 0.05).

Apoptosis analysis via caspases revealed that caspase-9 showed a significant reduction in the control group (CN) between weeks 12 and 16 (*p* = 0.0472). Treatment RO, on the other hand, resulted in a marked increase in caspase-9 compared to CN in week 16 (*p* = 0.0003). However, caspases-8 and -3 did not show significant variations over time in control groups or among different treatment types (*p* > 0.05) (Figure 3A–C) [20].

Calreticulin expression varied throughout the experiment. At week 12, group ART showed a significant reduction compared to CN (*p* = 0.0086), while groups RO and ARO exhibited significant increases compared to the control (*p* < 0.0001 for both). At week 16, only group RO maintained a significant increase compared to CN (*p* < 0.0001) (Figure 3D). Quantification of HMGB revealed a significant increase only in group RO at week 16, with a statistically significant difference compared to the corresponding control group (*p* = 0.0012) (Figure 3E). Analysis of IL-1β indicated significant changes between the control and RO-treated groups at week 12, suggesting that the intervention modulated the inflammatory response (*p* = 0.0075) (Figure 3F).

IFN-γ levels were significantly elevated in groups RO16 (*p* = 0.0117) and ARO16 (*p* = 0.0031) compared to CN16, highlighting a potential activation of the adaptive immune response (Figure 3G). Regarding GM-CSF, a significant increase was observed only in group RO at week 16 relative to the control (*p* = 0.0014) (Figure 3H). In contrast, IL-10 levels remained stable, with no statistically significant differences between control and treated groups at weeks 12 and 16 (*p* > 0.05) (Figure 3I), indicating that the treatments did not induce systemic immunosuppression.

## 3. Discussion

Due to a relatively high malignant transformation rate and the inability to predict lesion behavior, the clinical management of OPML remains a major clinical dilemma [21]. One method to overcome this challenge is chemoprevention, which can be of natural or synthetic origin and administered either topically or systemically [22]. Chemopreventive drugs should be easily administered to at-risk individuals with minimal toxicities to ensure acceptance and adherence to treatment. Toxicity is particularly important since patients may require therapy for prolonged periods [23]. In this study, the animals evaluated showed no signs of liver or kidney damage and maintained normal behavior throughout the experimental period.

The induction and treatment protocol were established based on the incidence of dysplasia and OSCC observed in our preliminary studies. These findings enabled the determination of optimal time points for analysis, allowing histopathological findings to be correlated with different stages of OL progression. By week 8, evidence of mild epithelial dysplasia was already present, indicating the onset of carcinogenesis. Mild dysplasia predominated at week 12, progressing to moderate and severe grades by week 16. Based on this timeline, experimental treatments were initiated at week 8, a strategic window to assess their chemopreventive potential against OL progression. Treatments were administered concomitantly with continuous exposure to the carcinogen 4NQO, simulating sustained consumption of carcinogenic agents such as tobacco. Subsequent euthanasias were conducted at weeks 12 and 16. Macroscopic and histopathological findings confirmed a gradual progression of lesions, with the duration of 4NQO exposure being directly proportional to the severity of tissue alterations. Consistent with our results, Hasina et al. demonstrated that administration of 100 μg/mL of 4NQO for 8 weeks induced dysplasia by week 12 and carcinoma by week 24, although without continuous exposure simulating tobacco use [24].

The histopathological findings over the weeks revealed distinct patterns of tumor progression among the experimental groups. By week 12, all treated groups showed a higher frequency of mild dysplasia compared to the control group, suggesting a potential early effect in containing lesion progression. This effect was especially pronounced in the RO-treated group, which exhibited the highest incidence of mild dysplasia (80%), indicating a potential protective effect associated with the compound’s antioxidant and immunomodulatory properties.

However, by week 16, a significant progression in lesion severity was observed across all groups. The control group already showed 50% carcinoma, while the ART group progressed to 40% carcinoma, and the ARO group demonstrated the highest malignant transformation rate (60%). This suggests that the combination of ART and RO, despite its immunomodulatory potential, may not have been sufficient to contain tumor progression. ART is a sesquiterpene lactone that contains an endoperoxide bridge, a structural feature critical for its biological activity. Upon activation by intracellular iron, the endoperoxide moiety generates reactive oxygen species, leading to oxidative stress, DNA damage, and induction of apoptosis in cancer cells [25]. Such mechanisms may account for the lower proportion of severe dysplasia observed in the ART group compared with the control group in our study. Notably, the RO group exhibited an increase in severe dysplasia (60%) but did not develop carcinoma by week 16. Analyses of this species reveal that, in addition to water, it is rich in calcium, β-sitosterol, α-carotene, ellagic acid, ellagitannins, and anthocyanins [26]. Anthocyanins, extensively studied in RO, are glycosylated with one or more sugar units, such as glucose, galactose, xylose, and arabinose, and are among the main bioactive compounds in this fruit. Ellagic acid, ferulic acid, and β-sitosterol have been shown to inhibit the growth of premalignant, malignant, and normal oral cell lines in vitro [27]. Similar results were described by Oghumu et al., who demonstrated that diets supplemented with 5% or 10% RO significantly reduced the incidence and multiplicity of OL, erythroplasia, and OSCC induced by 4NQO- in murine models [28].

Immunosuppression plays a critical role in the transformation of OL into OSCC [29]. Key immune cells involved in this process include T lymphocytes, dendritic cells, macrophages, and NK cells. In OSCC, CD8^+^ T-cell infiltration has been associated with better patient survival, whereas low expression correlates with increased perineural invasion [30]. ART and its derivatives are known to activate immune responses and enhance CD8^+^ T-cell production [31]. Dihydroartemisinin, a derivative of ART, has shown antitumor activity by increasing CD8^+^ T-cell infiltration in hepatocellular carcinoma [32]. However, in our study, CD8^+^ expression did not significantly increase in the ART group. In contrast, the ARO group showed elevated CD8^+^ levels throughout the experimental period, while the RO group exhibited a delayed response, with significant expression detected only at week 16. These findings are consistent with those of Ryan et al., who also observed increased CD8^+^ T-cell infiltration after RO supplementation. But in their study, the RO-enriched diet was initiated before 4NQO exposure, which may have enhanced its immunomodulatory effect [19].

As OL progresses to OSCC, a notable remodeling of the immune microenvironment occurs, marked by increased immunosuppressive cells and decreased antitumor mediators. This shift fosters immune evasion and creates a permissive environment for neoplastic growth, reducing the immune system’s ability to eliminate malignant cells [33]. In our study, significantly increased CD68 expression was observed in ART-treated animals at weeks 12 and 16, possibly indicating the recruitment of tumor-associated macrophages, particularly of the M2 phenotype, known for their pro-tumoral role. This suggests that despite ART’s therapeutic potential, the activation or persistence of a suppressive immune response may have contributed to tumor progression in these animals.

CD56 expression is also higher in OSCC compared to OL and is rarely observed in normal oral mucosa [34]. In our study, significantly reduced CD56 expression was observed in the ART group at week 12, a period during which less advanced lesions were also noted. This may suggest temporarily restrained tumor progression. However, given CD56’s association with NK cell activity, crucial in immune surveillance and elimination of transformed cells, its low expression may reflect a less responsive microenvironment, potentially contributing to the tumor progression observed in subsequent weeks. Together with the increase in CD68, this finding supports the notion that ART, under the tested conditions, failed to reverse the immunosuppressive profile characteristic of malignant OL transformation.

ART has well-established capabilities to induce apoptosis and ferroptosis in various cancer cells [35], while RO has been shown to activate the intrinsic apoptotic pathway through sequential activation of caspases-9, -3, and -7, leading to programmed cell death [36]. In our study, a significant increase in caspase-9 expression was observed in the RO group at week 16, indicating partial activation of the intrinsic apoptotic pathway. However, no significant changes were detected in caspase-3 or -8, which may suggest incomplete activation of the apoptotic cascade or the involvement of alternative cell death mechanisms modulated by RO. These findings support the chemopreventive potential of RO, although other mechanisms beyond classical apoptosis likely contribute to its anti-tumor effect.

At week 12, all experimental groups, except the ART group, which exhibited reduced calreticulin expression, showed elevated calreticulin levels compared to their respective controls. A similar increase was observed in the RO group at week 16. Calreticulin exposure on the surface of cells is a hallmark of immunogenic cell death, promoting phagocytosis and adaptive immune responses against tumor cells [37]. The elevated calreticulin levels in the RO group further support RO’s immunomodulatory profile, consistent with reports that RO regulates multiple immune components and inflammatory signaling pathways [38]. These include reducing reactive oxygen species production, restoring antioxidant enzyme activity, and inhibiting pro-inflammatory pathways like NF-κB and MAPK, which are implicated in tumor promotion [39]. Together, these results suggest that RO not only induces immunogenic cell death but also modulates tumor inflammation through multiple mechanisms.

Increased HMGB1 levels in the RO group at week 16 suggest that this compound actively influenced the tumor microenvironment. As a danger signal, HMGB1 is involved in tissue damage signaling and immune cell recruitment, and its elevation may indicate that RO promoted a controlled inflammatory response capable of enhancing immune surveillance [40]. Similarly, findings by Dutra et al. showed that oral squamous cell carcinoma cell lines (SCC-180) treated with cisplatin (CSP) at higher concentrations exhibited significantly reduced HMGB1 expression, whereas cells treated with lower concentrations of CSP, ART, or ART+CSP maintained levels similar to the negative control. These results reinforce the notion that sustained HMGB1 expression may be linked to immunogenic rather than cytotoxic responses. In this context, the increase in HMGB1 observed in the RO group may represent an immunomodulatory mechanism that favors immune activation and tumor surveillance, contrasting with the suppressive effect seen with high-dose conventional chemotherapy [41]. Supporting this, significant increases in IL-1β (RO, week 12) and GM-CSF (RO, week 16) were observed, cytokines associated with antigen-presenting cell activation and myeloid lineage differentiation. Furthermore, elevated IFN-γ levels in week 16 in both the RO and ARO groups reinforce RO’s immunomodulatory role, given IFN-γ’s central function in activating CD8^+^ T-cell cytotoxicity and curbing tumor progression. Collectively, these data highlight RO’s capacity to shape an immunologically active and chemopreventive tumor microenvironment.

## 4. Materials and Methods

### 4.1. Ethical Approval and Guidelines

This study was approved by the Research Ethics Committee of the Institute of Science and Technology, São José dos Campos/UNESP, under protocol No. 02/2021, and conducted in accordance with the Ethical Principles for Animal Experimentation. The experimental design followed a randomized, prospective, and controlled model, complying with the guidelines of Animal Research: Reporting In Vivo Experiments (ARRIVE) [42]. The sample size calculation was based on previous studies with similar methodology [41,43].

### 4.2. Animals and Housing Conditions

A total of 38 specific pathogen-free female C57BL/6J mice, aged 4 weeks and weighing approximately 20 g, were obtained from the Central Animal Facility at UNESP in Botucatu. The animals were maintained under controlled environmental conditions, with ad libitum access to food and water, at a temperature of 20 °C, 55% relative humidity, and a 12 h light/dark cycle. Each experimental group consisted of 5 animals per time point, while the control group consisted of 4 animals per period.

### 4.3. Oral Leukoplakia Induction with 4-NQO

To establish a reliable experimental model for the progression of OL to OSCC, a preliminary study was conducted in which different concentrations of 4NQO (50 µg/mL and 100 µg/mL) were tested in mice. The concentration of 100 µg/mL demonstrated greater efficacy in inducing histopathological alterations consistent with moderate to severe epithelial dysplasia and faster tumor progression and was therefore selected for the present study. After a 15-day adaptation period, lesion induction was initiated with the administration of 4-NQO (Sigma-Aldrich, catalog #N8141), diluted in 2-propanol (propylene glycol) (Sigma-Aldrich, catalog #N33539) to a stock solution of 4 mg/mL. It was prepared for a 15-day consumption period and stored at −20 °C. At the time of administration, the stock solution was diluted in filtered and autoclaved water to a final concentration of 100 μg/mL. Water bottles were wrapped in aluminum foil to protect the final solution from light, and both the solution and food were replaced twice weekly, with consumption measured at each replacement.

### 4.4. Experimental Treatments

Based on the preliminary findings, mild epithelial dysplasia began to appear in week 8. Therefore, treatment was initiated at this time point to target the early stages of lesion development. Experimental group ART received Artemisinin (ART) treatment, group RO received *Rubus occidentalis* extract (RO), and group ARO received a combination of both. ART (Sigma-Aldrich, St. Louis, MO, USA) was diluted in 1.6 mL of Dimethyl Sulfoxide (DMSO) (Neon Surface, SK, Canada) and administered via intraperitoneal injection at a dose of 85 mg/kg per week [44,45,46]. The RO (Berrihealth, Portland, OR, USA) extract was incorporated into pellets at a 5% concentration and prepared by Alquimia Farmácia de Manipulação (São José dos Campos, SP, Brazil) [47]. The RO-enriched chow was replaced twice a week, and its consumption was monitored. The control group (CN) received only 4-NQO without any treatment (Figure 1A).

The animals were closely monitored throughout the experiment for clinical signs such as changes in eating behavior, hair loss, signs of infection at injection sites, and weight fluctuations. Body weight was recorded biweekly using a semi-analytical scale. At weeks 12 and 16, animals were euthanized for sample collection. The tongues were excised and longitudinally bisected; one half was immediately stored at −80 °C in cryotubes for subsequent protein extraction, while the other half was fixed in 4% paraformaldehyde (Sigma-Aldrich, St. Louis, MO, USA) for histological and immunohistochemical analyses. Additionally, liver and kidney tissues were collected and fixed to assess potential systemic toxicity.

### 4.5. Histological Procedures

After fixation, tongues were embedded in paraffin, sectioned into 5 μm slices, and subjected to routine histological processing with H&E staining. Samples were analyzed using conventional light microscopy (Zeiss—Axioskop 40, Zeiss, Goettingen, Germany). Each sample was classified according to the 2022 World Health Organization (WHO) criteria. The grading of mild, moderate, and severe dysplasia and carcinoma was performed by two independent researchers (ISM and EK), who were blinded to treatment groups and time points, with Kappa testing used to assess interobserver agreement.

### 4.6. Immunohistochemistry Protocol

For immunohistochemical analysis, 3 µm thick tissue sections were prepared on silanized slides (Starfrost, Knittel, Braunschweig, Germany). The primary antibodies used were CD8, CD68 (Novus Biologicals, Centennial, CO, USA), and CD56 (Santa Cruz Biotechnology, Dallas, TX, USA). The immunohistochemistry methodology was based on Fernandes’s 2025 study [48]. Positive and negative controls were included in all analyses. The stained slides were digitized using the Pannoramic DESK device (3DHistech^®^, Budapest, Hungary), and the cellular markers CD8, CD56, and CD68 were quantified by directly counting the number of immunolabeled cells. CD8 and CD56 expressions were assessed on the cell membrane, whereas CD68 expression was evaluated on both the membrane and cytoplasm.

### 4.7. ELISA Assay

For ELISA testing, pre-sensitized antibody kits were obtained: HMGB, GM-CSF, IFN-γ, Caspase-9, and Calreticulin (Novus Biologicals, Centennial, CO, USA); Caspase-3, Caspase-8, and IL-10 (CLOUD-CLONE CORP, Uscn Life Science Inc., Wuhan, China); and IL-1β (Fine Test, Wuhan Fine Biotech Co., Ltd., Wuhan, China). The remaining frozen halves of the tongue were added to a solution containing a cOmplete™ Protease Inhibitor Cocktail tablet (Sigma-Aldrich, St. Louis, MO, USA), previously dissolved in T-PER™ Tissue Protein Extraction Reagent (Thermo Fisher, Waltham, MA, USA). The samples were then pooled to create a representative sample for each experimental group and diluted to a concentration of 60,000 pg/100 µL. The tests were conducted in sextuplicate, strictly following the specific recommendations of each manufacturer. Optical density readings were performed on a microplate reader at 450 nm (BioRad, Hercules, CA, USA).

### 4.8. Statistical Analysis

The variables in this study were tested for normality. Data analysis was performed using the statistical softwareGraphPad Prism 7 (GraphPad Software, Inc., Boston, MA, USA). Immunohistochemical data were analyzed using one-way ANOVA for parametric distributions and the Kruskal–Wallis test for non-parametric data, followed by Dunn’s multiple comparisons test with Bonferroni and Holm corrections. ELISA results were analyzed using unpaired t-tests. A *p*-value < 0.05 was considered statistically significant.

## 5. Conclusions

This study highlights RO, 5% in diet, as a promising agent for the chemoprevention of OL by positively modulating the immune microenvironment and preventing progression to carcinoma up to week 16. RO promoted the activation of immune and apoptotic pathways, including increased expression of caspase-9, calreticulin, HMGB1, IFN-γ, and GM-CSF, suggesting a protective immunomodulatory effect. In contrast, ART at 85 mg/kg/week alone did not show a significant effect and was associated with a more tolerogenic immune profile. The combination of both compounds also failed to prevent tumor progression. These findings underscore the potential of RO in preventing malignant transformation and emphasize the need for further studies to optimize treatment protocols and elucidate the underlying mechanisms.

## Data Availability

The datasets used and/or analyzed in the current study are available from the corresponding author upon reasonable request.
